# Polarization-resolved second harmonic generation imaging of human ovarian cancer

**DOI:** 10.1117/1.JBO.23.6.066501

**Published:** 2018-06-13

**Authors:** Kirby R. Campbell, Rajeev Chaudhary, Julia M. Handel, Manish S. Patankar, Paul J. Campagnola

**Affiliations:** a University of Wisconsin–Madison, Department of Biomedical Engineering, Madison, Wisconsin, United States; b University of Wisconsin–Madison, Laboratory for Optical and Computational Instrumentation, Madison, Wisconsin, United States; c University of Wisconsin–Madison, Department of Obstetrics and Gynecology, Madison, Wisconsin, United States

**Keywords:** collagen, ovarian cancer, second harmonic generation, polarization

## Abstract

Remodeling of the extracellular matrix in human ovarian cancer can be manifested in increased collagen concentration, changes in alignment within fibrils/fibers and/or up-regulation of different collagen isoforms. We used pixel-based second harmonic generation (SHG) polarization microscopy analyses to probe these molecular changes in human ovarian tissues [normal stroma, benign tumors, and high-grade serous (HGS) tumors] by: (i) determination of the α-helical pitch angle via the single-axis molecular model, (ii) collagen alignment within fibrils via SHG anisotropy, and (iii) chirality via SHG circular dichroism (SHG-CD). Pixel approaches are required due to the complex structure of the matrix that lacks a high degree of fiber alignment. The largest differences in the helical pitch angle were between normal stroma and benign tumors, consistent with gene expression showing the Col III isoform is up-regulated in the latter. The data were not consistent with up-regulation of Col III in HGS tumors as previous reports have suggested. The different tissues also displayed differing SHG anisotropies and SHG-CD responses, consistent with either Col III incorporation or randomization of Col I alignment within benign and malignant tumors. Additionally, the high-grade tumors displayed higher collagen concentration, where this desmoplasia is consistent with the higher fiber density in these tissues. These results collectively indicate that the fibril assemblies are distinct in all tissues, where these differences likely result from the synthesis of collagen rather than remodeling of existing collagen. Importantly, these analyses are label-free and interrogate subresolution collagen structure on intact tissues, without the need for conventional structural biology tools.

## 1 Introduction

In 2018, an estimated 22,240 new cases of ovarian cancer will be diagnosed and 14,070 women will die from this disease in the United States alone.^1^ A major impediment to ovarian cancer screening and treatment is a poor understanding of the tumor microenvironment (TME) in the ovary and fallopian tubes, from where recent evidence has shown many high-grade cases originate.^2^^,^^3^ Current diagnostic imaging modalities (e.g., MRI and CT) do not have sufficient resolution or sensitivity to probe TME structural changes. Additionally, serum methods lack sufficient specificity and sensitivity for effective screening of tumor progression.^4^^–^^8^ With the current repertoire of diagnostic modalities, only 15% of ovarian cancer cases are diagnosed while the disease is still localized to the ovary (stage I), suggesting the need for a new modality with the resolution and specificity to detect ovarian cancer before metastasis occurs. This is especially important for high-grade serous (HGS) tumors as these can metastasize while still microscopic.^9^

Although genetic changes associated with low- and HGS ovarian cancers have been widely explored^10^^,^^11^ the corresponding extracellular matrix (ECM) alterations that are not yet well known. We have previously taken steps in this direction by examining collagen architecture changes in different ovarian tumors using quantitative second harmonic generation (SHG) imaging and optical property measurements. For example, we have classified fiber patterns through two-dimensional (2-D) and three-dimensional (3-D) texture analysis,^12^^,^^13^ and probed fibril architecture through the analysis of the SHG directional pattern (forward–backward emission).^14^ Collectively, using a variety of metrics, we found quantifiable differences across tumor types (e.g., low grade, high grade, endometrioid, and benign tumors) relative to normal stroma.

The underlying molecular changes in collagen in ECM remodeling have received little attention and have been mainly limited to standard histology. Here, we report our efforts in probing collagen molecular aspects, specifically helical structure, concentration, and isoform distribution in normal stroma, benign tumors, and HGS ovarian tumors. This is important as, while the literature supports such modifications, the reports are highly qualitative, and moreover, specific forms of the alterations and their temporal sequences are not known.^15^ For example, it has been suggested that the total collagen content is lower in HGS cancer than in normal stroma; however, this collagen is hypothesized to be produced at higher rates with defective cross linking^15^ and thus turns over faster. This is in apparent contradiction to the desmoplasia seen in SHG imaging of collagen in HGS tumors.^14^ Similarly, while increases in minor isoforms of collagen type III (Col III) have been implicated in ovarian cancer, such studies used gene expression and immunofluorescence,^16^ and the abundance and assembly have not been uniquely quantified.^16^ Col III has also been reported to be up-regulated in benign tumors.^16^

To address these molecular changes, here we use several pixel-based polarization-resolved SHG methods including analysis of excitation and emission polarization as well as circular dichroism (SHG-CD) to probe macro/supramolecular structural aspects that further extend our differentiation capabilities beyond morphological metrics.^17^^–^^25^ Pixel-based analyses are necessary for tissues that do not have predominant fiber alignment, such as essentially all stromal tissues. We previously documented and validated these methods using the analysis of collagen self-assembled gels of known composition. For example, we have shown how to determine the net collagen α-helical pitch angle^26^ using a pixel-based analysis approach that combines the established single-axis model^26^ with the generic model developed by Brasselet and coworkers.^21^ We successfully applied this to differentiating Col I from Col III in fibrillar collagen gels.^21^ As remodeling can be in the form of different isoform expression or different assemblies in new collagen fibrils, other structural aspects such as the overall chirality or alignment of molecules within newly synthesized fibrils can also be different and are measurable by SHG-CD^23^ and SHG emission anisotropies,^21^ respectively.

We now apply these methods to *ex-vivo* human ovarian tissues, where we examined normal stromal tissues, benign serous tumors, and stage III and stage IV HGS tumors. Benign lesions are of significant interest as studies have shown that benign neoplastic epithelial tumors can progress into low-grade serous ovarian cancer.^27^^,^^28^

## 2 Materials and Methods

### 2.1 Human Samples

All ovarian tissues were obtained using a University of Wisconsin IRB-approved protocol from consented, deidentified patients undergoing surgical debulking treatment for ovarian cancer or benign gynecological conditions. This study examined normal stroma (n=4), benign tumors (n=3), stage III (n=3), and stage IV (n=3) HGS tumors. All samples were fixed in 4% formalin and cut into 100-μm-thick sections by a vibratome. These were then optically cleared by immersion in 50% glycerol for 12 h before imaging to reduce scattering-induced depolarization effects to afford the SHG polarization imaging. The samples were mounted within the same solution on slides, cover-slipped, and sealed with nail polish. All samples were each imaged at 10 separate locations to reach the appropriate statistical sample size.

### 2.2 Experimental Microscope Setup

The details of the SHG microscope have been described in detail by Chen et al.^29^ and Lien et al.^18^ and only briefly described here. The system consists of a laser scanning unit (FluoView 300; Olympus, Melville, New York) mounted on an upright microscope (BX61; Olympus, Tokyo, Japan), where the excitation source is a mode-locked Titanium Sapphire laser (Mira; Coherent, Santa Clara, California). Imaging was performed with a fundamental laser wavelength of 890 and 780 nm for linear polarization and CD, respectively, where the shorter wavelength for the latter provides greater sensitivity.^23^ Average powers at the focus were ∼30 to 50 mW using a 40×0.8 NA water immersion lens (LUMPlanFL; Olympus, Tokyo, Japan) and a 0.9-NA condenser. The resulting lateral and axial resolutions were ∼0.7 and 2.5 microns, respectively. The forward-directed SHG emission was collected using a photon-counting detector (7421 GaAsP; Hamamatsu, Hamamatsu City, Japan). The SHG wavelengths (445 and 390 nm) were isolated with the respective 10-nm-wide bandpass filters (Semrock, Rochester, New York). The excitation wavelength was confirmed using a fiber-optic spectrometer (Ocean Optics, Dunedin, Florida). About 512-×512-pixel images with a field-of-view size of 85  μm were acquired with scanning speeds of 2.71  s/frame with three-frame Kalman averaging. The power was controlled by an electro-optic modulator (ConOptics, Danbury, Connecticut) run by a custom LabVIEW program (National Instruments, Austin, Texas), interfaced with the FluoView scanning system using a data acquisition card (PCI-6024E; National Instruments).

Linear polarization was obtained using a half-wave plate to define the state entering the microscope and the desired linear rotation at the focal plane was achieved using a liquid-crystal rotator (LCR; Meadowlark Optics, Frederick, Colorado) mounted in the infinity space.^18^ Circular polarization is achieved with a quarter wave plate after the LCR, where left- and right-handed states are achieved with 90 deg of linear rotation by the LCR.^18^ The linear and circular polarization states were validated as previously described by imaging cylindrically symmetric giant vesicles.^18^^,^^23^ SHG signal anisotropy measurements further used a removable Glan–Laser Polarizer (analyzer) in a controlled motorized rotation stage (both from ThorLabs, Newton, New Jersey) before the detector. The polarization control was also run by a custom LabVIEW program interfaced to the FluoView scanning system.

For all analyses, we used fields of view that had approximately two-third collagen coverage to be consistent with other analyses we have performed. The pixel-based analysis is then performed over all pixels corresponding to collagen in the image (85×85 microns), and others are assigned not a number. The analysis is performed within 100 microns in thickness from the surface epithelium, as we have previously established is the location of the largest extent of remodeling.

### 2.3 Collagen Concentration Determination

Each of the ovarian tissue samples was homogenized in 1  mg/mL pepsin in 0.05-M acetic acid and incubated on a slow shaker for 72 h at 4°C. The supernatant was collected, and total collagen concentration was detected using a Sirius Red Collagen Detection Kit (catalog no. 9062, Chondrex, Redmond, Washington) in accordance with the manufacturer’s instructions. Tissues were analyzed from every specimen imaged by SHG polarization analysis.

### 2.4 Helix Pitch Angle and Anisotropy Analysis

Polarization-dependent measurements were performed as previously described,^21^ where images were taken every 10 deg through 180 deg of rotation for both the excitation and emission, or 361 images per optical section. Here, the method was applied to the four different groups of human ovarian tissues. The helix pitch angle is extracted^21^ by combining the pixel-based generic model^17^ with the single-axis molecular model.^26^ The SHG signal anisotropy was further determined on a pixel basis and also as a function of the laser polarization. The anisotropy, β, is reflective of the alignment of the dipole moments within the focal volume. The limiting cases are 0 and 1, representing totally random and perfectly aligned structures, respectively, and is calculated as

$$\beta (\theta )=\frac{I_{\mathrm{Par}}^{2\omega }(\theta )-I_{\mathrm{Perp}}^{2\omega }(\theta )}{I_{\mathrm{Par}}^{2\omega }(\theta )+2I_{\mathrm{Perp}}^{2\omega }(\theta )},$$ (1)

where $I_{\mathrm{Par}}^{2\omega }$ and $I_{\mathrm{Perp}}^{2\omega }$ represent the parallel and perpendicular SHG polarization response, respectively.

### 2.5 Second Harmonic Generation-Circular Dichroism

Nonlinear SHG-CD analysis was used to interrogate the chirality of the different groups of human ovarian tissues and has been described previously.^23^ Images were obtained 30-μm-deep into the tissues to avoid boundary effects. To account for variations in intensity in the different collagen mixtures, we report the normalized SHG-CD response defined as

$$I_{\mathrm{SHG}-\mathrm{CD}}=\frac{\left|I_{(2\omega )\mathrm{LHCP}}-I_{(2\omega )\mathrm{RHCP}}\right|}{[I_{(2\omega )\mathrm{LHCP}}+I_{(2\omega )\mathrm{RHCP}}]/2},$$ (2)

where $I_{(2\omega )\mathrm{LHCP}}$ and $I_{(2\omega )\mathrm{RHCP}}$ are the integrated pixel intensities of the SHG images for left-handed circular polarization (LHCP) and right-handed circular polarization (RHCP), respectively. This is calculated on a pixel basis, where we first set a threshold mask above the noise background. Absolute values were summed across the entire field of view as the sign of CD response will depend on fiber orientation.^23^

### 2.6 Statistical Analysis

For all polarization-resolved SHG metrics, one-way ANOVA tests followed by a least significance difference (LSD) tests were used to determine mean separation at the α=0.05 level. Two-sample t-tests were then performed on the average values for all tissue groups using the statistics toolbox in Origin 9.1 (OriginLab, Northampton, Massachusetts).

## 3 Results

### 3.1 Collagen Fiber Morphology and Concentration

**Fig. 1:**
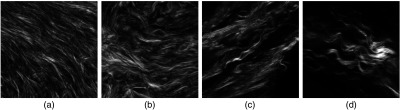
Representative SHG images of the four ovarian tissue classifications. The field-of-view size is 85 μm: (a) normal, (b) benign tumor, (c) stage III, and (d) stage IV.

Representative images of each of the four groups are shown in Fig. 1. In general, the SHG images of normal tissue are characterized by straighter, more cross-hatched collagen fiber morphology. Pathologically determined benign tumor tissues consist of clustered fibrous structures of a mixture of both straight and curvy features relative to normal stroma. Stage III and IV HGS tumor samples show long, wavy unidirectional fibers, where these overall patterns are well conserved within the patient population, although they appear more highly crimped in the latter samples. Collagen morphologies across different sampling regions do not vary significantly, as the analysis is limited to the highly dense collagen areas near the surface epithelium. We previously classified a spectrum of ovarian tumors by texture analysis^12^ and SHG physical attributes^14^ but did not delineate stage III and IV tumors, nor investigate the respective collagen abundance. The latter is important to determine if the change in morphology is related to desmoplasia (increased collagen deposition). These new aspects are delineated in the remaining sections.

**Fig. 2:**
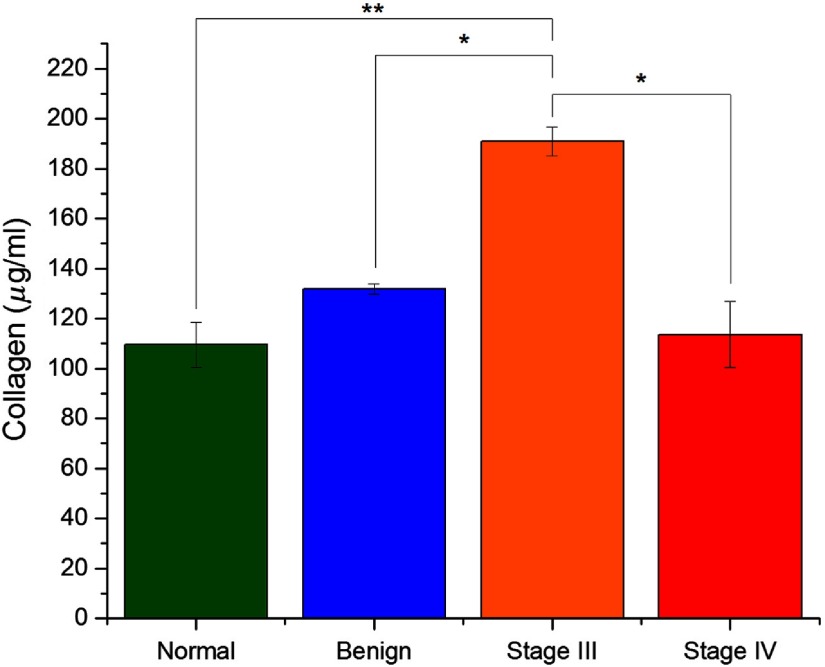
Quantified collagen amount for the four ovarian tissue classifications. Error bars represent standard error. * indicates p<0.05 and ** indicates p<0.01.

Collagen concentrations were then measured in each tissue using a Sirius Red detection kit and averaged for each sample group. The results of this assay are shown in Fig. 2. The measured concentrations were lowest in normal stroma and increased slightly in the benign tumors, although the difference was not significant. Stage III HGS tissues had significantly more total collagen than the other sample groups. This desmoplasia is consistent with our previous determination through SHG directionality and optical scattering measurements, both of which indicated increased collagen density.^14^ Interestingly, the stage IV total collagen levels were lower than benign and stage III tissue but slightly higher than normal stroma. The cause is unknown, but this could be due to degradation through increased protease activity in late stage disease following the initial desmoplastic response.

### 3.2 Second Harmonic Generation Linear Polarization Analyses

We next compared the SHG linear polarization responses (both excitation and signal analysis) of the four ovarian classification groups.

#### 3.2.1 Polarization dependence on the second harmonic generation intensity

**Fig. 3:**
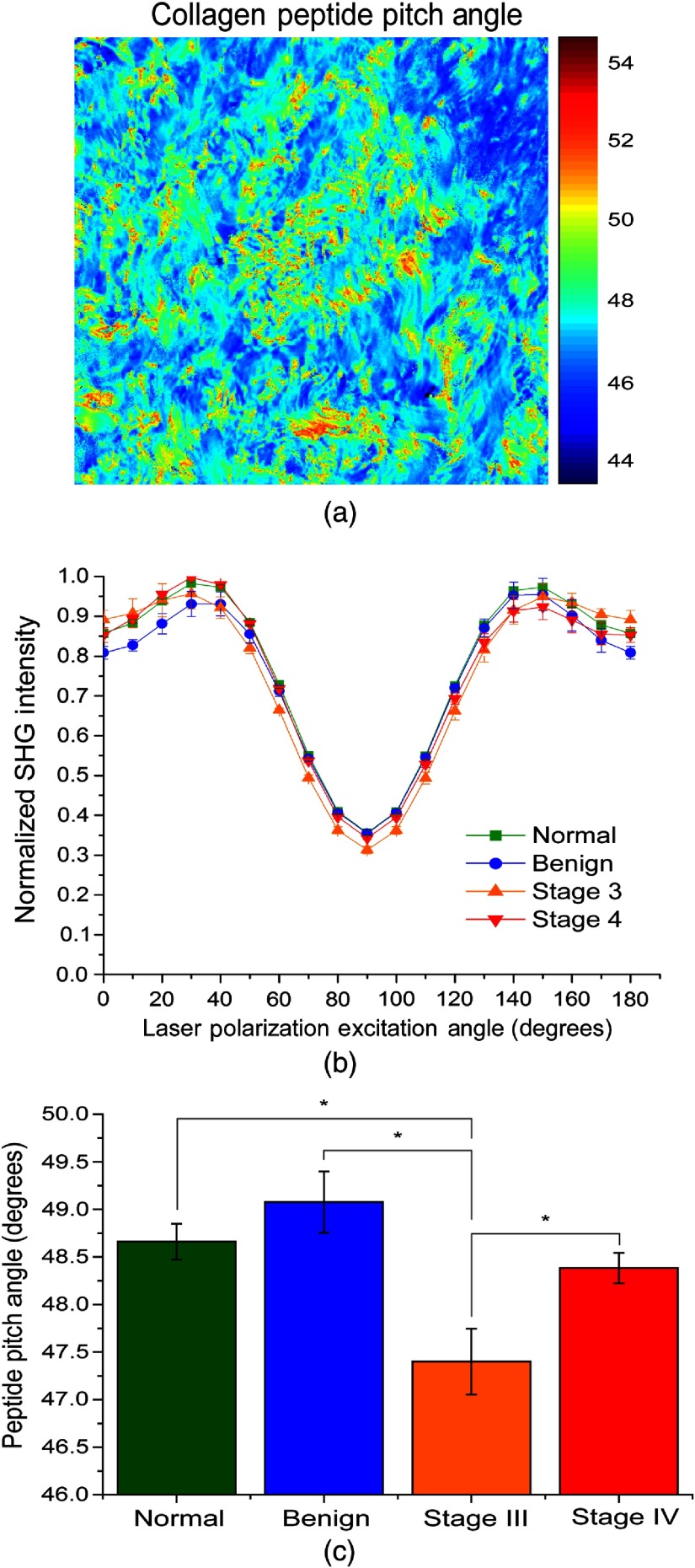
Pixel-based SHG polarization responses for the four ovarian tissue classifications. (a) Representative map of pitch angles for a stage III high-grade tissue, (b) reconstructed response based on the generic model, and (c) extracted pitch angles based on the single-axis molecular model from the reconstructed response with statistical differences (p<0.05). Error bars represent standard deviation.

Using pixel-based, linear polarization-resolved analysis, the effective pitch angle for each tissue class was determined by reconstructing the SHG intensity as a function of laser polarization dependence by fitting to the molecular model described in detail by Tilbury et al.^21^ A representative pixel map of pitch angles for a stage III tumor is shown in Fig. 3(a) and the reconstructed polarization response data for all tissues are shown in Fig. 3(b). The extracted results are shown in Fig. 3(c) and tabulated in Table 1. The significance categories in Table 1 are calculated by a one-way ANOVA test followed by an LSD test to determine mean separation at the α=0.05 level. We previously showed that the LSD of this measurement is 0.5 deg.^21^ The slight asymmetry at higher excitation angles is a small experimental artifact and does not affect data within four significant figures.

**Table 1:** Summary of SHG polarization-resolved methods, showing significance between groups.

SHG metric	Normal	Benign	Stage 3	Stage 4
Peptide pitch angle	48.7±0.2	49.1±0.3	47.4±0.3	48.4±0.2
Significance group	I	I	II	I
SHG anisotropy	0.71±0.02	0.55±0.02	0.64±0.02	0.63±0.01
Significance group	I	II	III	III
SHG-CD	0.289±0.006	0.250±0.005	0.265±0.005	0.261±0.006
Significance group	I	II	II	II

The extracted peptide pitch angle of the normal tissues is 48.66 deg and is similar to our previous measured results of tendon and 100% Col I gels of 49 deg and 49.11 deg, respectively.^21^ The net α-helical peptide pitch angle was largest for the benign tumor at 49.08 deg and could be attributed to increased Col III content, as this isoform has a higher pitch angle. The pitch angles of the stage III (47.4 deg) and stage IV (48.38 deg) tumor were both lower than the normal stroma, indicating that Col III is not increased in HGS cancers. We note that there is some heterogeneity of pitch angles within the pixel maps, and this will be the subject of further investigation.

#### 3.2.2 Second harmonic generation signal anisotropy

**Fig. 4:**
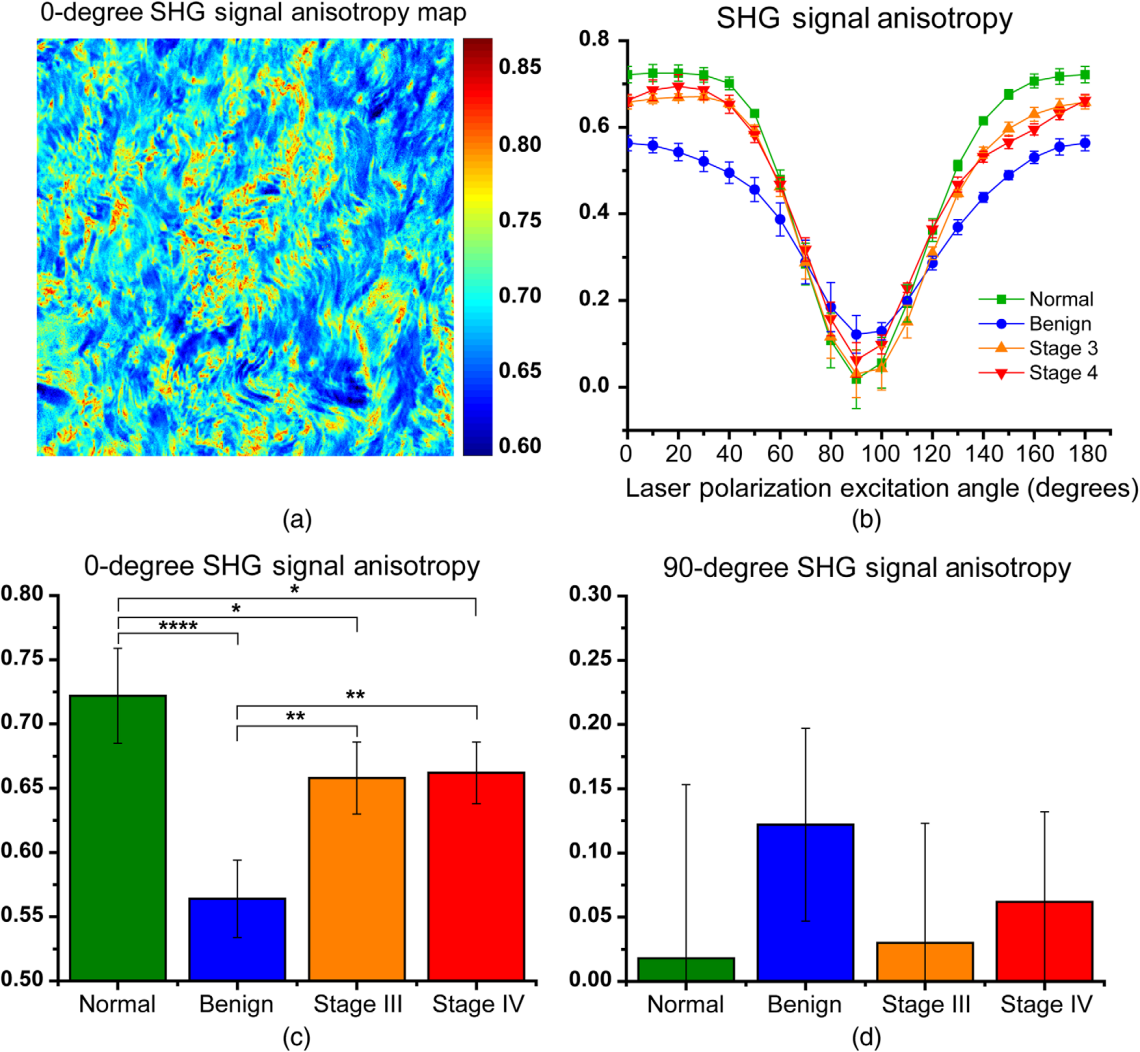
Pixel-based SHG signal anisotropy responses for the four ovarian tissue classifications depicting full curves. (a) Representative pixel map of 0 degree anisotropy for a stage III high-grade tissue and (b) reconstructed anisotropies at all excitation angles, and individual 0-deg and 90-deg angles shown in (c) and (d), respectively. * indicated p<0.05, ** indicates p<0.01, and **** indicates p<0.0001. Error bars represent standard deviation.

We next determined the SHG signal anisotropy within fibers in the four classes of ovarian tissues. Instead of acquiring the anisotropy at one angle as most commonly done,^30^ we use the full excitation/emission polarization dependence to determine the anisotropy at all excitation angles.^21^ A representative pixel map for a stage III tumor I with 0-deg excitation is shown in Fig. 4(a) and the excitation angle data for all tissues are plotted in Fig. 4(b). For simplicity, we focus the description on the anisotropy at 0- and 90-deg excitation, which for a linear fiber, corresponds to excitation parallel and perpendicular, respectively, to the primary axis. The resulting values with statistics are shown in Figs. 4(c) and 4(d) and tabulated in Table 1. For parallel excitation, the mean anisotropy value of the normal stroma was 0.71 and was the highest among the classes. This is physically reasonable as the collagen molecules align nearly on the long axis in a normal collagen fibril.^31^ Benign samples have a significantly lower anisotropy of 0.55 at 0 deg than normal ovarian tissue and higher at 90 deg. Stages III and IV had intermediate values at both 0- and 90-deg excitations. These results indicate the collagen molecules, either new Col I or Col III molecules do not lie parallel to the fibril axis in the benign and HGS tumors. We found analogous results in previous work analyzing Col I and Col III self-assembled gels, which were consistent with the two different isoforms comingling in the same fibrils.^21^ The anisotropies at 90 deg have the opposite trend for the tumors (i.e., higher anisotropy), and this was similarly found in our prior work on gels.^21^ We note that the error bars on the 90-deg excitation are large as the absolute signal levels were small and this precluded statistical significance; however, the over trend has structural consistency.

### 3.3 Second Harmonic Generation Circular Dichroism Analysis

**Fig. 5:**
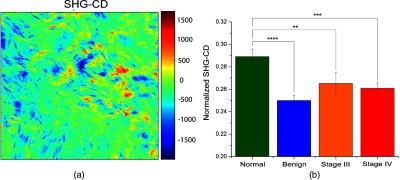
SHG-CD responses for the four ovarian tissue classifications. (a) Representative pixel map of SHG-CD for a stage III high-grade tissue and (b) normalized extracted SHG responses. ** indicates p<0.01, *** = p<0.001.

Next, we analyzed the same samples using the SHG-CD method described previously^23^ and averaged the measurements for each ovarian tissue group. A representative pixel map of SHG-CD for a stage III high-grade tissue is shown in Fig. 5(a), and the normalized extracted SHG responses are shown in Fig. 5(b) and tabulated in Table 1. We first note that the SHG-CD results closely match the trend of the anisotropy analysis. The mean SHG-CD response of the normal samples was the largest (0.289) and was significantly higher than the benign tumors (0.250), and the stages III and IV (0.261) tumors, respectively. This response is reflective of the net chirality within each pixel, and it would be expected that normal stroma would have the highest SHG-CD response, as the molecules align on axis in a normal fibril. Moreover, the value here is similar to our findings in tendon and Col I gels.^23^ The lower SHG-CD in the benign and high-grade tumors is reflective of either decreased alignment of molecules within fibrils or increased Col III expression. We note that this measurement is independent of the relative orientation of fibers within the image.^23^

## 4 Discussion

The etiology of ovarian cancer remains poorly understood, for example, even the primary location, i.e., the ovary itself or the fallopian tubes remains an open topic of research.^2^^,^^3^ Although many operative signaling pathways in ovarian cancer have been uncovered, underlying changes in the collagen in the ECM have not been well explored. For example, while we have documented structural changes in architecture by elucidating changes in fibril assembly through SHG creation physics^14^ and fiber organization by texture analysis,^12^ the molecular-level differences in collagen structure and concentration are unknown. The existing data are largely comprised of nonquantitative immunofluorescence imaging.^15^ To address this gap, we have implemented structural-based, polarization-resolved SHG analyses across a spectrum of ovarian tissues. Importantly, we have extensively validated these using well defined *in-vitro* models.^21^^,^^23^

It is known during malignant transformation, the epithelial cells undergo an epithelial to mesenchymal transitions,^32^^,^^33^ well as metalloproteinase upregulation (MMP-1, MMP-2, and MMP-9), both of which facilitate increased collagen remodeling.^34^^–^^36^ The results of the total collagen concentration assay performed in this study show significant increases in collagen density in the stage III tissues, consistent with our previous findings using other SHG metrics.^14^ Lower collagen density in the stage IV tissues may be the consequence of remodeling as faster collagen turnover has been suggested to occur in high-grader serous ovarian cancer.^37^ It is likely there are opposing effects on proteases on collagen content, as proteases can increase production of TGF-β as well as degrade the matrix. The temporal sequence of these processes is not known, but our findings lay the groundwork for future studies of these processes.

It has been suggested by immunofluorescence imaging that Col III is increased or turned over faster in ovarian cancer.^15^^,^^16^ We previously established that SHG linear polarization analysis could distinguish Col I and III by extraction of the differing pitch angles, where latter is larger by about 2 deg. The data here support benign tumors having higher Col III content based on the larger net pitch angle.^21^ However, the extracted pitch angles in the HGS tumors are not higher than the normal tissue, and are in fact lower, and do not support the conclusion of an increase in Col III concentration in these tissues. It remains possible that the Col III is increased but degraded rapidly by proteases and the effective Col I/Col III balance is not affected. We note that widely used commercially available Col III antibodies bind to both Col I and Col III (when tested in our *in-vitro* models) and thus are not highly specific. Due to this cross talk, we have not used them here for further validation.

The results of the SHG signal anisotropy and SHG-CD measurements revealed similar conclusions to each other. The collagen in normal stroma displayed the highest anisotropy and SHG-CD values, where these were both similar to those in self-assembled Col I gels and tendon. The lowest responses for each were found in benign tumors and are consistent with either an increase in Col III (in conjunction with the helical pitch angle) or altered alignment in Col I molecules within the fibril. Either would decrease the anisotropy or net chirality within the probed focal volume. Relative to normal tissue, the lower SHG anisotropy values in the HGS tumors may correspond to collagen molecules not being uniformly aligned within the collagen fibrils. Our previous work on ovarian cancer suggests that the collagen is newly synthesized as opposed to remodeled existing collagen,^14^ thus different alignments within newly made fibrils are possible. Similarly, the significant decrease in the SHG-CD response of these tissues is likely due to the same effect. An increase in Col III could lead to the same response; however, this is not consistent with the extracted pitch angles as discussed above.

We note that while the SHG anisotropy and SHG-CD measurements probe different aspects of structure (alignment of dipole moments versus net chirality within the focal volume), the respective findings are self-consistent in terms of structures of the tissue classes. Both these measurements indicate the benign tumors and high-grade tumors all have less organization than the normal stroma, although the underlying reasons are likely different. For example, the benign tumor and the high-grade tumor responses are consistent with increased Col III and protease activity, respectively. Although more work is needed to further characterize the specific molecular changes in structure, e.g., through mass spectrometry, we find statistically significant changes through these label free SHG metrics.

We note that a potential limitation of extending the SHG polarization methods to *in-vivo* applications is the significant polarization scrambling due to optical scattering, which can be further amplified by birefringence in collagenous tissues. Specifically, we have previously shown that SHG polarization signatures can be degraded in as few as two scattering lengths, or about 100 microns.^38^ For ovarian cancer, the largest remodeling is immediately beneath the surface epithelium, and shallow imaging (10 to 20 microns) may be sufficient. Here, to better sample the tissue, we needed to utilize optical clearing for these measurements as this process greatly preserves the polarization response through several hundred microns of tissue thickness.^38^ Moreover, these agents do not alter the underlying fibrillar structure and have shown great promise for *in-vivo* applications.^39^ Previous literature suggest that circularly polarized light is less prone to depolarization than linear polarization,^19^^,^^40^^,^^41^ and the SHG-CD could potentially be performed on somewhat thicker tissues than possible by the linear polarization analyses.

Although the data acquired in this study used forward emission detection schemes, a backward collection geometry could be used for the SHG polarization analyses, albeit with lower output signal. This is further possible as most tissues show similar features in the forward and backward directions. A common observation is that fibers detected in the backward direction can appear segmented, where this effect arises from the SHG coherence. The coherence length in the backward direction is shorter than the forward and as a consequence, these segments arise from destructive interference within the focal volume, where the same features are continuous fibers when viewed in the forward direction.^42^ We showed that the relevant structural aspect giving rise to the interference is the size and packing of the fibrils comprising the fibers. Thus, it is unlikely that the molecular aspects are different, as the structural aspects are the same.

## 5 Conclusions

We have shown that pixel-based polarization-resolved SHG imaging methods show great promise in delineating normal ovarian stroma, benign tumors, as well as different stages of HGS ovarian cancer by probing molecular changes in collagen. The linear polarization methods to extract the helical pitch angle and collagen molecular alignment and the SHG-CD to probe net chirality provide new complementary information to our previous work on the fibrillar hierarchy. Interestingly, our current findings indicate that Col III is not increased in HGS tumors, as previously suggested in the literature.^16^ Moreover, the collective findings that both benign tumors and high-grade tumors have different molecular assemblies within fibrils than those in normal ovarian stroma. These differences likely result from the synthesis of new collagen rather than remodeling of existing collagen. Although we cannot yet deduce the specific forms of the molecular changes, the results lay the groundwork for future spectroscopic efforts, e.g., FTIR and mass spectrometry. The ability to spatially and temporally map these species in tissue may provide new information on tumor dynamics and aid in determining whether these changes are associated with disease progression and patient outcome.
